# Erectile Dysfunction Among Male Hypertensives in a Tertiary Health Facility in South-West Nigeria

**DOI:** 10.5539/gjhs.v7n1p154

**Published:** 2014-08-22

**Authors:** Akinbode Samuel Fafiolu, Ayodeji Matthew Adebayo, Temilola Olufunmi Akande, Olubankole Olutosin Akinboboye

**Affiliations:** 1 Department of Family Medicine, Federal Medical Centre, Owo, Ondo State, Nigeria; 2 Department of Preventive Medicine and Primary Care, College of Medicine, University of Ibadan, Ibadan, Nigeria; 3 Department of Medicine, College of Medicine, University of Ibadan, Ibadan, Nigeria; 4 Federal Medical Centre, Owo, Ondo State, Nigeria

## Abstract

**Introduction:**

Erectile dysfunction (ED) has been associated with hypertension and some other chronic diseases. There are few studies on ED in Nigerian male hypertensives and ED appears to be under-reported. We sought to determine the prevalence of ED among hypertensive and normotensive men and to assess the association of demographics, hypertension, antihypertensive medications and other risk factors with erectile function.

**Methods:**

A comparative cross sectional study was conducted among male adult hypertensive and normotensive patients attending the outpatient clinic of a tertiary hospital in South-West Nigeria. A systematic random sampling method was employed for the selection of respondents. Participants were interviewed using a semi-structured questionnaire to document socio-demographic data, medical history, social history and degree of ED. Demographic and anthropometric characteristics was obtained from all participants. The International Index of Sexual Health Inventory for men (SHIM) was used to determine the presence and severity of ED. Association between categorical independent variables and erectile function were tested using Chi square and the predictors of erectile dysfunction determined with binary logistic regression model at 5% level of significance.

**Results:**

A total of 202 male patients completed the study (101 with established hypertension and 101 normotensives who served as comparative group). The mean age of the respondents was 49.74 ± 16.6 years. A total of 133 (65.8%) respondents had ED in varying severities while 34.2% had normal erectile function. Mild to moderate ED occurred in 29.7% while 36.1% had severe ED. On bivariate analysis, prevalence of ED was higher among hypertensives (75%) than normotensives (56.9%) and this was statistically significant, p = 0.007. On multivariate analysis, the only significant risk factor for ED was age. The elderly aged ≥ 65 years (OR: 2.9; 95% CI: 1.03–8.35; p = 0.04) and those aged 46–64 years (OR: 2.9; 95% CI: 1.38–6.53; p = 0.006) were 3 times each more likely to have erectile dysfunction compared with those aged ≤ 45 years.

**Conclusion:**

This study revealed that erectile dysfunction was prevalent in both hypertensive and normotensive population studied and that this was significantly worse with increasing age. A higher proportion of hypertensives compared to normotensives had erectile dysfunction. We recommend that all men presenting to a physician should have routine evaluation for ED so as to recognise it early and reduce its effects.

## 1. Introduction

Erectile dysfunction (ED) is defined as the consistent inability to achieve or sustain an erection for satisfactory sexual activity. It is a global health problem found in men of all ages though it is said to be commoner in persons with hypertension. Prevalence rates for ED vary according to characteristics of the population studied and the method used to assess erectile function. In Nigeria varying prevalence rates of ED ranging from 41.5% to 57.4% have been reported in apparently normal men (Idung et al., 2012; Fatusi et al., 2003; Adebusoye et al., 2012; Shaeer et al., 2003).

Elderly men are the worst affected by ED as various chronic disorders are more common in this age group and these are associated with elevated rates of ED. In the Massachusetts Male Aging Study (MMAS) the risk of ED was four times higher in men aged 60-69 years than for men aged 40-49 years and ED was inversely related to education and income (Johannes et al., 2000). ED can be organic or psychogenic. Organic ED can be due to vascular, neurogenic, anatomic or endocrine causes. ED has been considered an early marker of cardiovascular risk that could indicate the presence of vascular disease (Roth et al., 2003).

Hypertension and its treatment are known to produce sexual dysfunction in males. ED is a common complaint in association with hypertension though it is frequently under-reported and under-diagnosed. Hypertension is said to be a common cause of vasculogenic ED which may cause arterial insufficiency which decreases the perfusion pressure and arterial flow to the penis. Conditions that share the same risk factors with ED such as diabetes, hyperlipidemia, obesity and smoking are also associated with arterial insufficiency.

Several authors have reported a significant association between hypertension and ED (Nunes et al., 2012; Okeahialam & Obeka, 2007; Shaeer et al., 2003). Hypertension is common and many drugs used in its treatment can cause ED. The under-reporting of ED may suggest that it is not searched for, nor volunteered. We sought to determine the prevalence of ED among hypertensive and normotensive men and to assess the association of demographics, hypertension, antihypertensive medications and other risk factors with erectile function.

## 2. Methods

This was a descriptive cross-sectional study carried out in a tertiary hospital in South-West Nigeria. The Hospital provides primary, secondary and tertiary levels of healthcare. After initial screening, a systematic random sampling method was used to select two hundred and two sexually active men (101 males with established hypertension and 101 normotensives who served as the comparative group) who attended the family medicine clinic and met the inclusion criteria. Persons recruited as subjects were sexually active males aged 21 years and above who had been attending the family medicine clinic for treatment of hypertension. Controls were persons with normal blood pressure and no previous history of hypertension who presented to the family medicine clinic for other medical conditions. Persons with diabetes mellitus, hyperlipidaemia, renal disease, heart failure, stroke, history of previous urological surgery and penile injury were excluded from the study. Ethical clearance was obtained from the Health Research Ethics Committee of the Hospital and written informed consent was obtained from all participants.

Information was collected from participants using a semi-structured questionnaire to document socio-demographic data, medical history, social history, degree of ED and physical and laboratory measurements. Demographic and anthropometric characteristics were obtained from all participants. Weight was measured in kilograms using a beam type scale without the subject wearing heavy clothes or shoes. The height was measured in meters without the subject wearing shoes, cap or headgear and standing with the back to the measuring rod and looking straight ahead. Body mass index (BMI) was calculated as the weight in kilograms divided by the height in meters squared. Blood pressure was measured with a mercury sphygmomanometer after the subject had rested for about five to ten minutes in a quiet room. Hypertension was defined as blood pressure of ≥ 140/90 mmHg on at least two occasions or if the patient was on antihypertensive medications. The International Index of Sexual Health Inventory for men (SHIM) was used to determine the presence and severity of ED. The SHIM is an instrument that has been validated for assessment of erectile dysfunction. It is a widely used screening scale for ED with internal consistency of 0.9. The reliability of this instrument using Cronbach’s Alpha in our study was 0.92. In the SHIM, presence of ED was defined as SHIM score of less than 22. ED was classified as severe (SHIM score 1–7), moderate 8–11, mild to moderate 12–16, mild 17–21, and normal erectile function 22–25.

In our study, the SHIM score was modified to give three categories because an overlap was observed in the categories. Moderate scale (8–11) was combined with severe (1–7) to indicate “severe ED’’ (1–11), mild to moderate scale (12–16) was left as “mild to moderate ED” (12–16), while mild scale (17–21) was combined with normal (22–25) to indicate “no ED’’ (17–25). For bivariate and multivariate analysis, two categories were used. Scores of 1–16 were categorized as “erectile dysfunction’’ while scores of 17–25 were categorized as ‘’no erectile dysfunction’’.

Venepuncture was done under aseptic condition and blood was collected for estimation of fasting plasma glucose and fasting total cholesterol. Persons who met the criteria for diagnosis of hypercholesterolemia (fasting total cholesterol > 6.0 mmol/l and criteria for diabetes mellitus (fasting plasma glucose ≥ 7 mmol/l) were excluded from the study. Data was entered into a computer and analysed using SPSS version 15. Data were reported as means, standard deviation (SD) and percentages. Association between independent and dependent variables were explored using Chi square for categorical qualitative variables and t-test for quantitative variables. Predictors of erectile dysfunction were elicited using binary logistic regression model. The level of significance was set at 5%.

## 3. Results

A total of 202 male patients completed the study. The demographic profile of the respondents is presented in Table 1. The mean age of the respondents was 49.74 ± 16.6 years. Ninety-six (47.5%) of the respondents were 45 years old and below while 106 (52.5%) were 46 years and older. Forty-eight percent of the patients took alcohol while 12.9 % smoked cigarettes. 27.2% were either overweight or obese.

**Table 1:** Socio-demographic characteristics of respondents

Variable n=202	Frequency	Percentage
**Age group in years**	
**≤45**	96	47.5
**46-54**	66	32.7
**≥65**	40	19.8
**Ethnicity**	
**Yoruba**	162	80.2
**Others**	40	19.8
**Marital status**	
**Ever married**	176	87.1
**Never married**	26	12.9
**Highest level of education**	
**No formal**	15	7.4
**Primary**	47	23.3
**Secondary**	79	39.1
**Tertiary**	61	30.2
**Religion**	
**Christianity**	171	84.7
**Islam**	29	14.3
**Smoking status**	
**Yes**	26	12.9
**No**	176	87.1
**Alcohol intake**	
**Yes**	97	48
**No**	105	52
**BMI status**	
**Underweight**	8	4
**Normal**	139	68.8
**Overweight**	45	22.2
**Obese**	10	5.0

A total of 133 (65.8%) respondents had ED in varying severities, 34.2% had normal erectile function (no ED), 29.7% had mild to moderate ED while 36.1% had moderate to severe ED. Prevalence of ED was higher among hypertensives (75%) than among normotensives (56.9%) and this was statistically significant, p=0.007 (Table 2).

**Table 2:** Association between respondents’ characteristics and erectile dysfunction

	Erectile dysfunction n (%)	No erectile dysfunction n (%)	Chi square	P value
**Age group (years)**				
**≤45**	49 (51.0)	47 (49.0)		
**46-64**	52 (78.8)	14 (21.2)		
**≥65**	32 (80.0)	8 (20.0)	17.8	*<0.001
**Marital status**				
**Ever married**	117(66.5)	59 (33.5)		
**Never married**	16 (61.5)	10 (38.5)	0.25	0.62
**Highest level of education**				
**No formal**	11 (73.3)	4 (26.7)		
**Primary**	36 (76.6)	11 (23.4)		
**Secondary**	53 (67.1)	26 (32.9)		
**Tertiary**	33 (54.1)	28 (45.9)	6.59	0.086
**Alcohol intake**				
**Yes**	69 (71.1)	28 (28.9)		
**No**	64 (61.0)	41 (39.0)	2.32	0.13
**Tobacco intake**				
**Yes**	20 (76.9)	6 (23.1)		
**No**	113 (64.2)	63 (35.8)	1.63	0.20
**BMI**				
**Normal**	99 (67.3)	48 (32.7)		
**Overweight & Obese**	34 (61.8)	21 (38.2)	0.54	0.46
**Hypertensive**				
**Yes**	75 (75.0)	25 (25.0)		
**No**	58 56.9	44 43.1	7.39	*0.00
**Antihypertensive use**	
**Yes**	43 (78.2)	17 (21.8)		
**No**	90 (61.2)	57 (38.8)	5.12	*0.024

* Statistically significant.

A significant increase in the prevalence of ED with age was noted. The prevalence increased from 51% among those 45 years old and below to 80 % among those that were 65 years and above.

The association between ED, demographic factors, hypertension and obesity are summarized in table 3. On multivariate analysis, the only significant risk factor was age. In terms of age, men 45 years old and above were almost 3 times more likely to have ED (odds ratio 2.9) compared to men younger than 45 years (p value 0.006). Age was the only important predictor of ED on multivariate analysis as smoking, alcohol use, hypertension, antihypertensive use and overweight/ obesity were not significantly associated with ED in this study population (Table 3).

**Table 3:** Predictors of erectile dysfunction in respondents

Variable	Odds ratio	95% confidence interval	P value
**Age group**			
**≤45**	1.000		
**46-65**	2.998	1.376-6.531	*0.006
**≥65**	2.933	1.030-8.349	*0.044
**Highest level of education**			
**No formal**	1.000		
**Primary**	1.625	0.770-3.433	0.203
**Secondary**	1.460	0.564-3.780	0.436
**Tertiary**	1.447	0.361-5.790	0.602
**Smoking**			
**No**	1.000		
**Yes**	1.296	0.436-3.855	0.641
**Alcohol use**			
**No**	1.000		
**Yes**	1.593	0.816-3.111	0.173
**Hypertension**			
**No**	1.000		
**Yes**	1.601	0.722-3.554	0.247
**Anti- hypertensive use**			
**No**	1.000		
**Yes**	1.100	0.416-2.911	0.847
**BMI**			
**Normal**	1.000		
**Overweight & obese**	0.685	0.339-1.383	0.291

* Statistically significant.

## 4. Discussion

Our study demonstrated an overall prevalence of ED of 65.8% amongst respondents. The prevalence was higher than the rates of between 43.8% and 57.4% reported in previous studies in Nigeria (Fatusi et al., 2003; Afolayan & Yakubu, 2009; Olugbenga-Bello et al., 2013). A multi-centered report on the prevalence of erectile dysfunction among men attending primary care clinics in three countries showed that age-adjusted prevalence rates of ED was 57.4% in Nigeria, 63.6% in Egypt, and 80.8% in Pakistan (Shaeer et al., 2003).

The increased prevalence of ED in our study could be attributed to several factors. Increasing level of modernisation and awareness of treatment options for ED, individuals are sensitised and more able to acknowledge this problem to their health care provider and are more willing to participate in research relating to ED.

It could also be due to the fact that it is a hospital-based study, and though we excluded patients with some known risk factors for erectile dysfunction, we may not have totally eliminated persons with other risk factors for ED [such as use of certain medications associated with ED (antidepressants, finasteride, anxiolytics, neuroleptics, NSAIDs and muscle relaxants), obesity, lower urinary tract symptoms] (Shamloul & Ghanem, 2013). Another explanation could be in the selection of respondents as most were relatively older (over half were above 45 years of age than those reported in some other studies (Fatusi et al., 2003; Olugbenga-Bello et al., 2013).

Our study found a higher prevalence of ED in hypertensives compared to normotensives [75% versus 56.9% respectively (p=0.007)]. Okeahialam in a study done in Jos, Nigeria reported ED prevalence rates of 47.2%, 52.4% and 5.4% respectively in newly diagnosed treatment-naïve hypertensives, hypertensives on treatment and normotensive individuals. Several authors have reported significant association between hypertension and ED though it is not clearly known how hypertension causes ED. One theory is that there is a low production of nitric oxide by the arteries (including the penile arteries) in hypertensives. Nitric oxide is essential for increased production of cyclic guanosine monophosphate in the penis causing relaxation of the corpora cavernosa and leading to an erection (Burnett, 2006). Another reason that could be adduced is the progression of atherosclerosis in hypertensives which can contribute to ED (Kloner & Speakman, 2002).

Another explanation could be in our selection of patients as our respondents were relatively older (over half of our respondents were over 45 years old) than in some of the studies above (Fatusi et al., 2003; Olugbenga-Bello et al., 2013).

On bivariate analysis our study showed a significant association between hypertension and ED. However on further analysis using binary logistic regression, hypertension was not found as a significant risk factor even though those that were hypertensive were 1.6 times more likely to have ED than the normotensives (Table 3).

This study showed that age was the only important predictor of ED. Those aged ≥ 65 years and 46-64 were 3 times each more likely to have erectile dysfunction than those 45 years and below. Several studies have reported strong association between age and ED (Johannes et al., 2000; Wagle et al., 2012; Hwa et al., 2012). This could be as a result of the association between aging and many chronic disorders which are organic causes of ED such as hypertension, diabetes and dyslipidemia. Aging is also associated with decreased total serum and bioavailable testosterone concentrations. In the Massachusets male aging study complete impotence increased from 5% among men 40 years of age to 15% among men 70 years and older (Johannes et al., 2000). Some studies in Nigeria also found a significantly increasing prevalence of ED with age (Fatusi et al., 2003; Adebusoye et al., 2012). BMI, alcohol and cigarette intake were not significantly associated with ED in this study though some studies have found an association between intensity of cigarette intake and ED (Cho et al., 2003; Pourmand et al., 2004) and between obesity and ED (Chao et al., 2011).

This study had some limitations: we did not assess the type of ED in the respondents, the effect of antihypertensives could not be ascertained due to the relatively small sample size and the instrument used in assessing ED was subjective as it was by reported symptoms which could have been over or under reported.

In conclusion, we found that ED was prevalent in both hypertensive and normotensive population studied and that this was significantly worse with increasing age. A higher proportion of hypertensives compared to normotensives had ED. Some modifiable cardiovascular risk factors like obesity, alcohol and cigarette ingestion were not significantly associated with ED.

We recommend that all men presenting to a Physician should have routine evaluation for ED so as to recognise it early and reduce its effects. Since ED could be associated with hypertension and other medical illnesses, the finding of ED should prompt the doctor to do a thorough screening of the patient especially for cardiovascular diseases. Older men should be evaluated and offered proper management of the disease as age was found to be a significant predisposing factor. We also recommend a larger Community-based study in this environment to possibly establish the causal effects of hypertension on ED in this population.
